# Comparing the effects of acute alcohol consumption in germ-free and conventional mice: the role of the gut microbiota

**DOI:** 10.1186/s12866-014-0240-4

**Published:** 2014-09-16

**Authors:** Canesso MCC, Lacerda NL, Ferreira CM, Gonçalves JL, Almeida D, Gamba C, Cassali G, Pedroso SH, Moreira C, Martins FS, Nicoli JR, Teixeira MM, Godard ALB, Vieira AT

**Affiliations:** Department of Physiology and Biophysics, Institute of Biological Sciences, Universidade Federal de Minas Gerais, Belo Horizonte, Brazil; Department of General Biology, Institute of Biological Sciences, Universidade Federal de Minas Gerais, Belo Horizonte, Brazil; Department of General Pathology, Institute of Biological Sciences, Universidade Federal de Minas Gerais, Belo Horizonte, Brazil; Department of Microbiology, Institute of Biological Sciences, Universidade Federal de Minas Gerais, Belo Horizonte, Brazil; Department of Biochemistry and Immunology, Institute of Biological Sciences, Universidade Federal de Minas Gerais, Belo Horizonte, Brazil; Department of Pharmacology, Institute of Biomedical Sciences - ICB-1, São Paulo University, São Paulo, Brazil

**Keywords:** Alcohol, Germ-free, Microbiota, Dysbiosis, Fiber, Liver, Gut, Inflammation

## Abstract

**Background:**

Increasing evidence suggest that the gut microbiota plays an important role in liver pathology after acute alcohol intake. The aim of our study was to investigate the roles played by commensal bacteria in alcohol-induced liver injury and in the dysbiosis caused by alcohol intake in germ-free mice, as well as the possibility of protection against alcohol-induced injuries in animals fed a high-fiber diet. For these purposes, germ-free and conventional mice were submitted to acute alcohol intake, consisting of administration of ethanol in their drinking water for 7 days, with a higher dose of alcohol administered on day 7.

**Results:**

There was no liver injury after alcohol consumption, and there was less neutrophil infiltration and lower pro-inflammatory cytokine levels (CXCL-1/KC and interleukin (IL)-6) in the liver in germ-free mice compared with alcohol-fed conventional mice. Additionally, conventionalization of germ-free mice with intestinal contents from alcohol-fed conventional mice induced injury and inflammation in both the liver and the intestine, suggesting that alcohol intake successively caused a perturbation of the intestinal microbiota (dysbiosis) and liver injury. Finally, previous treatment with a high-fiber diet decreased liver injury and gut permeability in alcohol-fed conventional mice.

**Conclusions:**

In conclusion, the results of the present study provide evidence that the gut microbiota plays an important role in alcohol-induced liver injury, apparently through dysbiosis of the intestinal microbial ecosystem caused by alcohol intake. Furthermore, treatment with a high-fiber diet can counteract hepatocyte pathology and gut leakage and thus could be a promising therapeutic option.

**Electronic supplementary material:**

The online version of this article (doi:10.1186/s12866-014-0240-4) contains supplementary material, which is available to authorized users.

## Background

Alcohol consumption, which is associated with severe hepatic damage (e.g., fibrosis and cirrhosis) [1,2], is one of the major causes of chronic liver disease in Western countries [3]. Of the many factors that contribute to the pathogenesis of alcohol-induced liver injury, gut-derived bacterial products seem to play a central role in inducing steatosis and inflammation. In particular, a high level of lipopolysaccharide (LPS) is found in the blood of patients with chronic alcohol consumption, and this phenomenon is related to a range of factors, including a predominant change in the composition of the intestinal microbiota [4,5]. This endotoxin may favor alteration of the balance of normal bacteria, hindrance of beneficial commensal bacteria, increase intestinal permeability by producing inflammatory mediators and alcohol metabolites in the intestine, and contribute to dysfunction of the intestinal epithelial cells and alteration of tight junctions [6]. It has been observed that increased intestinal permeability facilitates the translocation of microbial products, such as LPS, from the intestinal lumen to the extra-intestinal organs [7]. After these gut-derived bacterial products translocate from the gut to the liver, they interact with TLR4 on Küpffer cells, leading to production of TNF-α and oxidative stress, which cause hepatocellular damage [3].

Another factor that contributes to the severity of alcoholic liver disease (ALD) is the infiltration of the liver by neutrophils. Studies have shown that the systemic activation of neutrophils by pro-inflammatory cytokines, chemokines, complement factors, and other biologically active molecules (e.g., platelet-activating factor) can lead to the migration of neutrophils to the liver, where they kill sensitized hepatocytes by releasing inflammatory mediators [8].

The intestinal microbiota has emerged as an important factor in triggering inflammatory responses. Because products of the unbalanced normal intestinal microbiota are important mediators of the hepatic injury induced by excessive alcohol intake, alterations in the composition of the intestinal microbiota may explain its role in the pathogenesis of liver injury. In fact, recent studies have confirmed that the intestinal microbial composition is altered in alcoholism [9,10]. This alteration (dysbiosis) could be one of the factors that may exacerbate inflammatory responses, even systemically, after alcohol ingestion. Thus, restoring the normal composition of the intestinal microbiota may be an alternative treatment for reducing the hepatic injury caused by excessive alcohol intake. Probiotic, prebiotic and high-fiber diets are several of the possible ways to intervene in the intestinal microbial ecosystem.

In the present study, it was hypothesized that alcohol consumption alters the composition of the gut microbiota, causing dysbiosis, and that this alteration plays a role in the inflammatory response induced in the liver. To investigate this hypothetical role of the microbiota, germ-free mice were compared with their conventional counterparts in an acute alcohol intake model. In addition, the treatment of mice with a high-fiber diet was evaluated as a possible means to decreasing dysbiosis of the microbiota and the consequent liver injury.

## Results

### Germ-free mice show reduced liver pathology after alcohol administration compared with conventional mice

**Figure 1:**
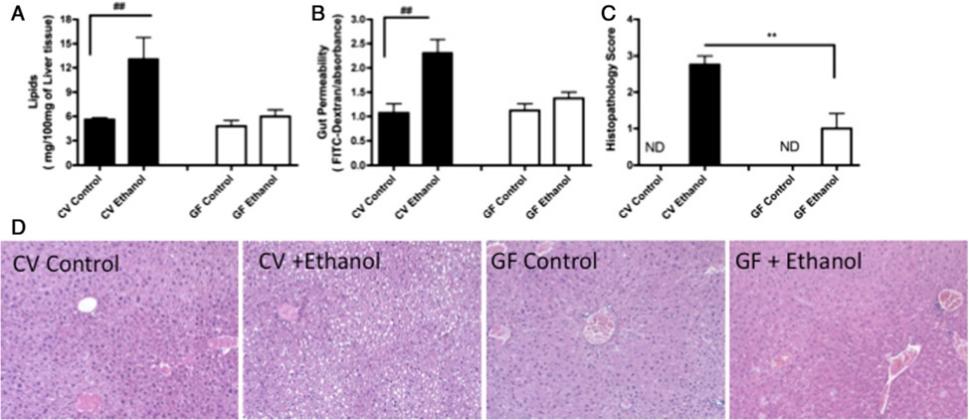
**Influence of alcohol treatment on lipid accumulation in the livers of conventional and germ-free mice.** Germ-free mice that underwent alcohol treatment did not show any lipids in their livers at 9 hours after the high dose of alcohol was administered on day 7 compared with the conventional mice, which showed an increase in their liver lipid content **(A)**. Gut permeability was also evaluated by FITC-dextran fluorescence in the serum **(B)**. Liver damage score **(C)**. Representative pictures of the livers of the control and alcohol-fed (+ethanol), conventional (CV), and germ-free (GF) mice. Control CV and GF mice: absence of lesions; CV mice + ethanol: diffusely distributed lipid microvacuoles; GF mice + ethanol: focal and discrete hepatic microvacuoles **(D)**. Hematoxylin and eosin (H&E) staining (200X). The results are the mean ± SEM (n = 5-7/group). This experiment is representative of at least three experiments. ^##^p < 0.01, conventional vs. conventional treated with alcohol.

Lipid accumulation in the liver is an important characteristic of hepatic pathology that is known as steatosis. To evaluate hepatocyte damage in the mouse groups, we measured the lipid levels in the liver. We observed that germ-free mice that were subjected to alcohol treatment showed no altered liver lipid content nine hours after the high dose of alcohol was administered on day 7. In contrast, the livers of the conventional mice showed increased lipid levels (p < 0.01) at the same time (Figure 1A). Moreover, germ-free mice treated with alcohol did not show alteration in gut permeability compared with control germ-free mice. In contrast, conventional mice increased intestinal permeability after excessive alcoholic intake (Figure 1B). Score of liver damage histopathology confirmed that ethanol consumption induced the formation of lipid microvacuoles that were diffusely distributed in the conventional mice (CV + Ethanol) and focally distributed in the germ-free mice (GF + Ethanol) (Figure 1C, D). There was no difference in hydric consumption between the groups tested (Additional file 1).

**Figure 2:**
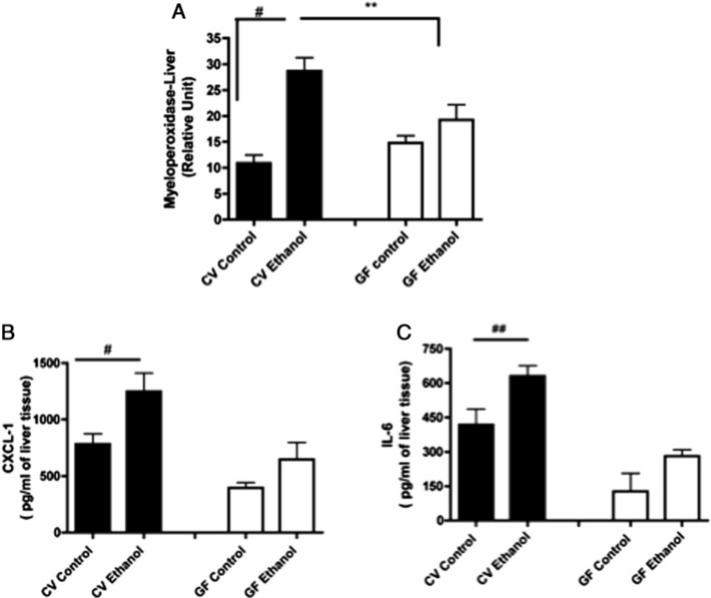
**Influence of alcohol treatment on inflammatory parameters in the livers of germ-free and conventional mice.** The number of neutrophils that had accumulated in the liver was estimated using an MPO assay **(A)**. Levels of the pro-inflammatory cytokines CXCL-1/KC and IL-6 in the liver (**B** and **C**, respectively). The cytokine levels were measured by ELISA. The data represent the mean ± SEM (n = 5-7/group). ^#^p < 0.05, ^##^p < 0.01, 9 hours vs. 0 hours after the high dose of alcohol was administered; ^**^p < 0.01, conventional vs. germ-free mice.

The profiles of the inflammatory response in the livers of conventional and germ-free mice were also examined. Neutrophils are important inflammatory cells that contribute to ALD, as they infiltrate the liver after being activated by pro-inflammatory cytokines and kill sensitized hepatocytes [8]. To evaluate neutrophil accumulation in the liver, we used a myeloperoxidase (MPO)-based activity assay. MPO is the most abundant enzyme in neutrophils, and it has been shown to be a useful and reliable marker for neutrophil infiltration [11]. We observed an increased level of neutrophil infiltration in the livers of the conventional mice (p < 0.05) after the final high dose of alcohol was administered on day 7 compared with the conventional control group (Figure 2A). In contrast, no difference was observed in the neutrophil accumulation in the livers of the germ-free mice after alcohol treatment (Figure 2A). In agreement with the liver lipid and neutrophil data, alcohol intake also increased the levels of the pro-inflammatory cytokines CXCL-1/KC and interleukin (IL)-6 in the livers of the conventional mice (p < 0.05 and p < 0.01, respectively) but did not affect these levels in the germ-free mouse livers (Figures 2B-C). Together, these findings indicate that the indigenous microbiota plays an important role in hepatocyte pathology after alcohol consumption.

### Alcohol consumption causes intestinal bacterial overgrowth and dysbiosis

Given that the inflammatory response after alcohol intake was more prominent in the small intestine (data not shown), we sought to determine whether alcohol intake leads to intestinal dysbiosis. Therefore, we collected the contents of the small intestines of control conventional mice and conventional mice treated with alcohol to quantify the CFUs in different media. Our results showed that alcohol consumption increased the number of cultivable bacteria observed, based on the CFUs counted, and thus increased the total number of bacteria compared with the numbers in the control mice (p < 0.05) (Table 1). Interestingly, the largest difference between the groups was observed in the numbers of enterobacteria (p < 0.001).

**Table 1:** **Quantitative analysis of microbial populations in conventional mice treated with or without alcohol utilizing the feces cultivation-dependent method** (**CFU/g of feces)**

**Selected media**	**Selected microorganisms**	**Experimental groups**	
		**Control**	**Ethanol**
**MacConkey**	Enterobacteria	1.7 x 10^8^ ± 1745	1.9 x 10^9^ ± 1500 (***)
**Heart Infusion Broth with Azide**	*Enterococcus*	1.0 x 10^5^ ± 0.68	4.0 x 10^8^ ± 3986 (*)
**Supplemented Blood Media (Cultivated in O**_**2**_ **)**	Total Aerobic Bacteria	5.6 x 10^8^ ± 2534	2.6 x 10^9^ ± 6699 (*)
**Supplemented Blood Media (Cultivated in CO**_**2**_ **)**	Total Anaerobic Bacteria	6.4 x 10^8^ ± 3640	2.1 x 10^9^ ± 3987 (*)
**MRS**	Lactic Acid Bacteria	4.9 x 10^8^ ± 2593	2.7 x 10^9^ ± 6071 (*)

(The data represent the mean ± SEM, *p < 0.05; ***p < 0.001).

### Conventionalization of germ-free mice with intestinal contents from alcohol-fed conventional mice induces inflammation in the small intestine and the liver

**Figure 3:**
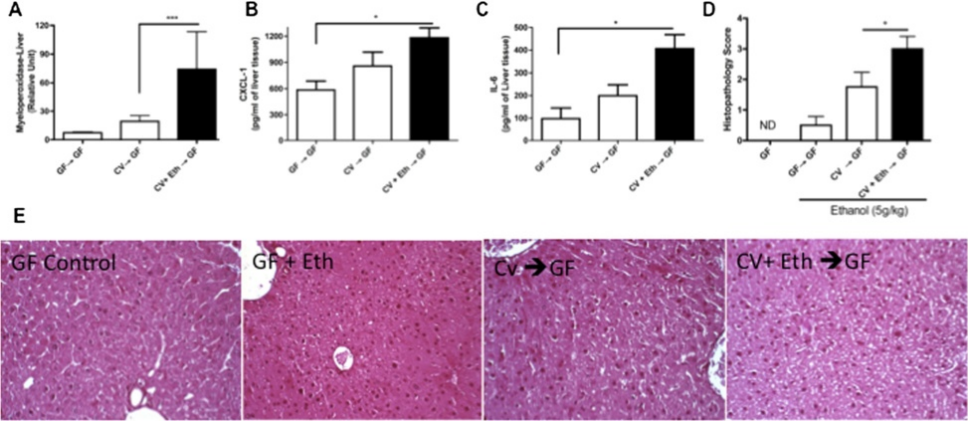
**Influence of alcohol treatment on inflammatory parameters in the livers of germ-free mice conventionalized with intestinal contents from conventional mice.** The number of neutrophils that had accumulated in the liver was measured using an MPO assay **(A)**. Pro-inflammatory cytokine (CXCL-1/KC **[B]** and IL-6 **[C]**) levels in the liver. The cytokine levels were measured using ELISA. Liver score **(D)**. Representative pictures of the livers of the germ-free mice (GF Control), the germ-free mice that underwent alcohol treatment (GF + Ethanol), the germ-free mice colonized with intestinal contents from the conventional mice (CV → GF), and the germ-free mice colonized with intestinal contents from the conventional mice treated with alcohol (CV + Eth → GF) **(E)**. PAS staining (200X). The data represent the mean ± SEM (n = 5-7/group). ^*^p < 0.05; ^***^p < 0.001.

To determine whether the commensal microbiota or a dysbiotic alcohol-altered microbiota is important to liver injury, germ-free mice were administered the intestinal contents of other germ-free mice (GF → GF) or were conventionalized with intestinal contents from conventional mice treated with (CV + Eth → GF) or without (CV → GF) alcohol. These animals then underwent the same alcohol treatment as described above. In the CV → GF group, compared with the GF → GF group, increases in neutrophil accumulation (Figure 3A) and in the levels of the pro-inflammatory cytokines CXCL-1/KC (Figure 3B) and IL-6 (Figure 3C) were observed in the liver after treatment with alcohol. Interestingly, these increases were even more pronounced in the CV + Eth → GF group compared with the CV → GF group. Histopathological score evaluation confirmed these data in both CV → GF and CV + Eth → GF mice, showing increased multifocal cytoplasmic microvacuolation than in ethanol-treated GF → GF mice (Figure 3D, E).

**Figure 4:**
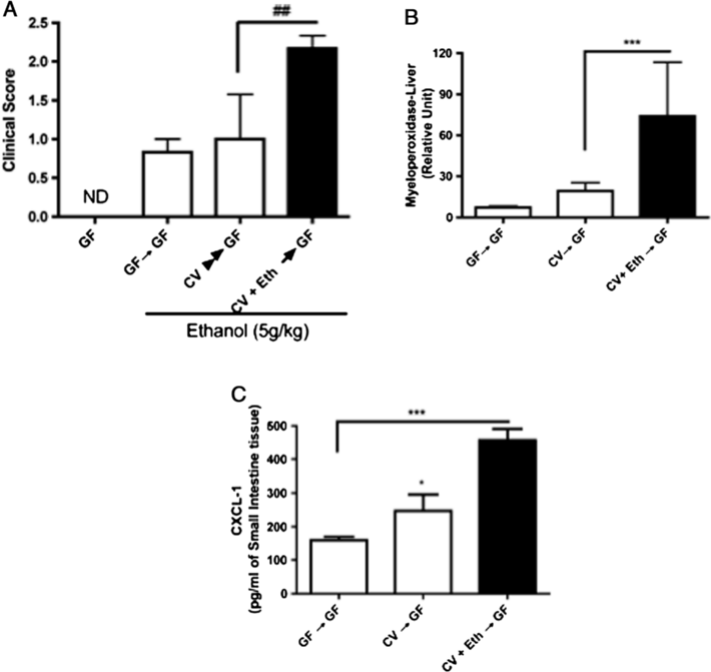
**Influence of alcohol treatment on the clinical score and inflammatory parameters in the small intestines of conventionalized mice.** Clinical score **(A)**. The number of neutrophils that accumulated in the small intestine was measured using an MPO assay **(B)**. Levels of the pro-inflammatory cytokine CXCL-1/KC in the small intestine **(C)**. The cytokine levels were measured by ELISA. The data represent the mean ± SEM (n = 5-7/group). ^*^p < 0.05, ^**^p < 0.01 and ^***^p < 0.001.

The greater effect of the dysbiotic, alcohol-altered microbiota on liver injury observed above was further observed at the small intestinal level, as demonstrated by the higher values obtained for the clinical score, neutrophil accumulation and CXCL-1/KC levels in CV + Eth → GF mice compared with GF → GF and CV → GF mice (Figures 4A, 4B and 4C, respectively). Altogether, these data suggest that excessive alcohol intake leads to a dysbiotic microbiota, which is crucial in promoting inflammation, gut leakage, and liver injury [9].

### Fiber treatment reduces gut permeability and protects conventional mice from liver injury after acute alcohol consumption

**Figure 5:**
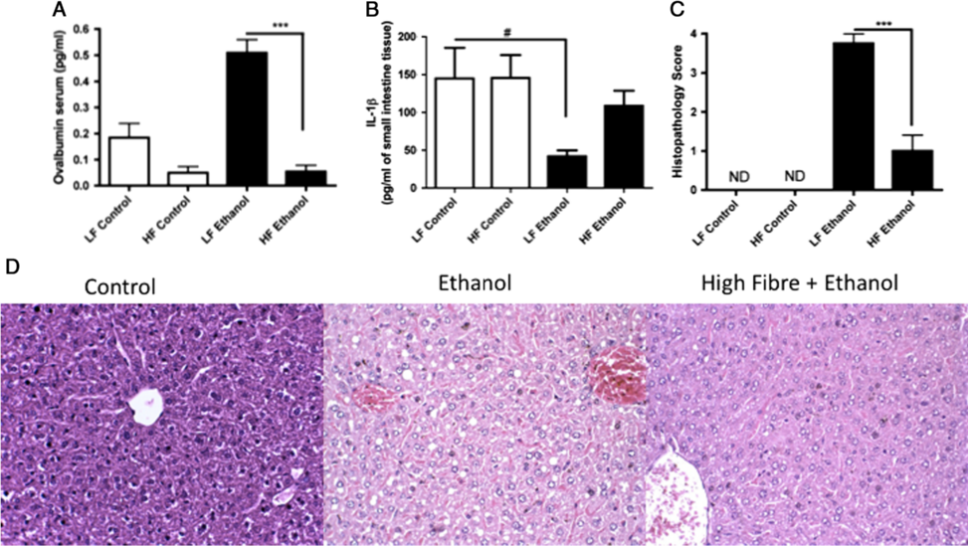
**Influence of alcohol treatment on intestinal permeability in conventional mice treated with experimental diets.** The ovalbumin levels in the serum were determined as a measure of gut permeability **(A)**. The IL-1β levels in the small intestine **(B)** were measured by ELISA. Liver damage score **(C)**. The CV mice (control) had no hepatic lesions, the ethanol-fed CV mice presented diffuse hepatic microvacuolation, and the alcohol-fed CV mice that received a high-fiber diet exhibited discrete microvacuolation **(D)**. H&E staining (200X). The data represent the mean ± SEM (n = 5-7/group). ^#^p < 0.05, the conventional mice that received a low-fiber diet and underwent alcohol treatment (LF + Ethanol) vs. the conventional mice received a low-fiber diet and were not treated with alcohol (LF Control); ^***^p < 0.001, the conventional mice that were treated with alcohol and received a low-fiber diet (LF + Ethanol) vs. the conventional mice that were treated with alcohol and received a high-fiber diet (HF + Ethanol).

As a high-fiber diet is known to promote the growth of beneficial bacteria, to control possible dysbiosis in the gut, and to protect individuals from inflammation [12,13], conventional mice were treated with a high-fiber diet to assess the possible protective effect on gut leakage after alcohol consumption. Treatment with a high-fiber diet (HF + Ethanol) decreased intestinal permeability after excessive alcohol intake, which was not observed in mice treated with a low-fiber diet before alcohol intake (LF + Ethanol) (Figure 5A). As IL-1β is known to be critical in the protection of epithelial cells [14], its levels were also measured in the small intestines of all of the groups tested (Figure 5B). The levels of this cytokine were similar in the groups receiving the LF and HF diets but decreased sharply in the LF + Ethanol group. Consumption of a high-fiber diet also restored IL-1β levels to normal values in the small intestines of the alcohol-fed animals. Liver score histology evaluation confirmed these data in HF + Ethanol mice, which showed reduced microvacuole density in the liver (implying a reduction in lipid accumulation in the liver) compared with the density in the LF + Ethanol group. The latter group presented a high density of lipid microvacuoles in the liver, which is characteristic of early-stage steatosis (Figure 5C, D).

## Discussion and conclusions

ALD constitutes a large proportion of liver disease worldwide. Despite extensive investigation over the past few decades, we still do not fully understand the mechanism of this disease and therefore lack an effective therapy. The results obtained in the present study, comparing germ-free and conventional mice submitted to acute alcohol intake, confirm that the gut microbiota plays an important role in alcohol-induced liver injury. In particular, in the absence of intestinal microbiota, there is no inflammation and no liver disease. Moreover, alcohol-induced liver injury seems to be associated with dysbiosis of the intestinal microbiota, most likely caused by alcohol intake. Treatment with a high-fiber diet prevented this alcohol-induced liver injury and gut leakage, providing an interesting alternative for treating the consequences of alcoholism.

Previous studies have shown the gut-liver axis is an important pathway in the development of liver injury after excessive alcohol consumption [1-3]. The two main mechanisms of gut barrier disruption involve the metabolites of alcohol, such as acetaldehyde, and a change in the composition of the gut microbiota caused by alcohol [15,16]. These alterations allow the passage of gut-derived endotoxins into the liver, leading to liver injury [17]. Here, we showed that in the absence of intestinal microbiota, no liver injury was observed after alcohol feeding in mice. Furthermore, these mice did not show any changes in their levels of the inflammatory cytokines/chemokines CXCL-1/KC and IL-6. These results indicate that alcohol metabolites alone are not sufficient for the development of hepatocyte pathology and that the presence of an altered microbiota and its products is also necessary. Additionally, LPS has been reported as a key bacteria-derived product that induces inflammation in alcoholic patients [7,17], as it is found at high levels in the circulation of alcoholics with liver disease [4,5,18]. Thus, treatment with an endotoxin (LPS)-neutralizing protein significantly suppresses the alcohol-induced elevation of plasma endotoxin levels and liver injury and inhibits TNF-αproduction [19]. These studies suggest the importance of the altered gut microbiota and gut permeability in increasing LPS levels in the plasma during ethanol consumption.

The balance of gut bacteria, intestinal permeability and hepatocyte function appears to be critical for maintaining normal homeostasis of the gut-liver axis. Alcohol consumption affects the composition of the gut microbiota, induces bacterial overgrowth (Gram negative) and allows more bacterial products to travel from the intestine to the liver [17,20]. Dysbiosis is thus an important factor in the pathogenesis of ALD in patients with leaky intestines. Here, we show that following alcohol consumption, there is a significant alteration in the gut microbiota, which can be transferred from one animal to another, as demonstrated by the worse liver injury observed in the CV + Eth → GF mice compared with the CV → GF mice. This injury was characterized by increased hepatocyte pathology and higher levels of inflammatory mediators in CV + Eth → GF mice.

The alteration in the gut microbiota consists of an increase in the total number of bacteria observed, mainly in the Enterobacteriaceae group, of which *Escherichia coli* is a representative species. Interestingly, this group of bacteria is associated with several pathological conditions in the gastrointestinal tract, such as inflammatory bowel disease [21-23]. These results align with the literature, which has described alterations in the composition of the microbiota after alcohol intake in humans and mice [6,24]. Interestingly, treatment with the probiotic *Lactobacillus*, a Firmicutes bacterium whose number is decreased in alcohol-fed mice, protects mice from alcohol-induced liver injury [6,25], thereby demonstrating the importance of restoring the composition of the normal microbiota in alcohol-induced disease.

Multiple factors likely contribute to the changes that occur in the intestinal microbiota during the development of ALD. These changes include small intestinal dysmotility [26], changes in gastric acid secretion [27], and alterations in the innate immune response in the intestine. This situation justifies attempts to restore eubiosis, which might in turn restore intestinal homeostasis [24] to treat liver disease. It is well established that diet affects the composition of the gut microbiota [12,28,29], so this approach may be a way to manipulate the microbial ecosystem. Prebiotics are defined as food ingredients that specifically promote the growth of beneficial bacteria and consequently promote both homeostasis in the gut and good health [30]. Soluble fiber has certain characteristics of prebiotics (they pass intact through the small intestine and are metabolized in the colon by components of the local microbiota) but are used only by a specific group of beneficial bacteria. Nevertheless, we analyzed the effects of a high-fiber diet enriched with pectin on alcohol-derived liver disease. The results showed that the animals fed a high-fiber diet showed less liver injury and lower intestinal permeability after alcohol consumption compared with the animals that received a low-fiber diet. The beneficial effect of fiber ingestion could be due to prevention and/or reversal of the effects of alcohol on barrier integrity and/or to compensation for dysbiosis of the microbiota. The latter effect could restore the normal commensal bacteria, such as Firmicutes bacteria, which are known to produce short-chain fatty acids (SCFAs). In addition to these possibilities, another putative mechanism that may explain fiber’s benefits involves an increase in IL-1β production in the intestine. Although IL-1β is classically known as a pro-inflammatory cytokine, recent studies have shown that it is also involved in epithelial repair [28]. Dietary fiber can be broken down into SCFAs, such as acetate, propionate and butyrate, by the normal intestinal microbiota to obtain energy [28,30]. These SCFAs bind to epithelial cells, inducing the activation of inflammasomes. Inflammasomes are cytoplasmic multi-protein complexes that sense microbial products and are composed of NLRs, adapter proteins, and procaspase-1, which trigger IL-1β and IL-18 maturation [31]. The data in the present study show that the IL-1β levels in the intestine were increased in animals fed a high-fiber diet and that this cytokine possibly protected these mice against alcohol-induced liver injury. These results align with those of a recent study in which increased levels of IL-1β were observed in the liver in association with hepatic regeneration after alcohol-induced injury [15]. Moreover, SCFAs stimulate the release of mucin, which is important for mucus secretion, providing physical protection against invasion by pathogenic bacteria and preventing an increase in gut permeability [32]. Thus, our data are the first to show that dietary fiber can preserve gut permeability after alcohol intake, providing an interesting alternative therapy for alcoholic patients.

In summary, the present study suggests that the indigenous intestinal microbiota is involved in liver injury due to high alcohol consumption. Apparently, changes in the composition of the gut microbiota (dysbiosis) induce an increase in gut permeability and subsequent trafficking of bacterial products to the liver, causing damage. In addition, the data show that ingestion of a high-fiber diet decreases gut permeability and liver injury after alcohol intake and thus could be an interesting therapy for alcoholic patients.

## Methods

### Mice

Eight- to ten-week-old female germ-free NIH Swiss mice were obtained from Taconic Farms (Germantown, NY, USA) and maintained in flexible plastic isolators (Standard Safety Equipment, McHenry, MD, USA) using classical gnotobiology techniques [33]. Conventional NIH Swiss mice are derived from germ-free matrices and are considered to be conventional only two generations after conventionalization. Water and a commercial autoclavable diet (Nuvilab, Nuvital, Curitiba, PR, Brazil) were sterilized by steam and administered *ad libitum*. For experiments, animals were maintained in micro-isolators (UNO Roestvaststaal, The Netherlands) located in a ventilated animal caging system (Alesco Ltd., Campinas, SP, Brazil) with controlled lighting (12 h light, 12 h dark), humidity (60-80%) and temperature (22 ± 1°C). Experiments using germ-free mice were conducted under aseptic conditions to avoid infecting the animals [34]. All procedures complied with the standards stated in the Guide for the Care and Use of Laboratory Animals (Institute of Laboratory Animal Resources, National Academy of Sciences, Bethesda, MD, 1996) and were conducted under conditions approved by the local animal ethics committee (CETEA/UFMG, 119/2012).

### Experimental design

Groups of 5–7 animals were used to separately evaluate the influences of microbiota, conventionalization and high-fiber treatment. For determination of the influence of microbiota, the following four groups of mice were tested: GF Control: germ-free control; GF + Ethanol: germ-free treated with alcohol; CV Control: conventional control; and CV + Ethanol: conventional treated with alcohol. For determination of the influence of conventionalization, the following four groups were tested: GF: germ-free control; GF → GF: germ-free treated with alcohol; CV → GF: germ-free conventionalized with intestinal contents from conventional control; and CV + Eth → GF: germ-free conventionalized with intestinal contents from conventional treated with alcohol. Finally, for determination of the influence of fiber, the following four groups were tested: LF: conventional fed a low-fiber diet; HF: conventional fed a high-fiber diet; LF + Ethanol: conventional fed a low-fiber diet and treated with alcohol; and HF + Ethanol: conventional fed a high-fiber diet and treated with alcohol.

### Alcohol treatment

The alcohol treatment protocol included administering ethanol to the mice (10% vol/vol) in their drinking water for 7 days, with additional oral gavage with a higher dose of alcohol (5 mg/kg) on day 7. The mice were then sacrificed at different time points after the oral gavage [35].

### Conventionalization

The process of conventionalization of germ-free mice with microbiota from conventional mice was performed as previously described [33,36]. Briefly, the intestinal contents were removed from the large intestine of germ-free mice (GF → GF), conventional mice (CV → GF), and conventional mice that had undergone the alcohol treatment (CV + Eth → GF) and were homogenized in saline (10%) in an anaerobic chamber (Forma Scientific Company, Marietta, OH, USA) with an atmosphere of 85% N_2_, 15% H_2_ and 5% CO_2_. The homogenates were then administered by oral gavage to germ-free mice. Fourteen days later, these animals were subjected to the alcohol treatment protocol, as described above. To assess whether there was adequate colonization of the germ-free mice, fecal samples were cultured using a thioglycollate test [36].

### Fiber treatment

To assess the effect of fiber treatment, fourteen days prior to ethanol administration and during the entire experimental period, conventional animals were supplied either with the AIN93 [37] modified as a special high-fiber diet using enrichment with 10% pectin-soluble fiber (HF) or with a low-fiber diet (LF) (Additional file 2).

### Assessment of clinical score

The mice were monitored for nine hours after the final high dose of ethanol was administered via oral gavage. They were then left alone in a cage for 10 minutes to obtain fecal samples for analysis. Fecal blood was tested using Hemoccult test cards (INLAB-Diagnostica, São Paulo, Brazil). Graded scores were given as follows: 0 = feces with a normal consistency and no blood in the fecal blood test, 1 = feces with a smooth consistency and no blood in the fecal blood test, 2 = feces with a pasty consistency and a low level of blood in the fecal blood test, and 3 = liquid feces and a high level of blood in the fecal blood test.

### Quantification of neutrophil accumulation

The extent of neutrophil accumulation in the liver tissue was measured by assaying MPO activity, as previously described [11]. Briefly, liver tissue was removed and snap-frozen in liquid nitrogen. Upon thawing and processing, the tissue was assayed for MPO activity by measuring the change in the optical density at 450 nm using tetramethylbenzidine. The results were expressed as the neutrophil index, which denotes the activity of MPO related to casein-elicited murine peritoneal neutrophils processed in the same manner.

### Measurement of cytokine and chemokine concentrations in the small intestine and liver

The levels of IL-1β, IL-6, and CXCL-1/KC were measured in small intestine and liver tissue samples using commercially available antibodies according to the manufacturer’s instructions (R&D Systems, Minneapolis, MN, USA).

### Gut permeability assessment

Gut permeability was evaluated in the animals by administering one dose (80 mg/animal) of ovalbumin (Sigma, St. Louis, MI, USA) 90 minutes before the high dose of alcohol on day 7. Sera were then collected from these mice, and ELISAs were performed to detect the ovalbumin level in the blood. The level of ovalbumin was directly proportional to the gut permeability. FITC-dextran (MW 4000; Sigma, St. Louis, MI, USA) gavage was also used to assess gut permeability. More specifically, after 4 hours of fasting, the mice were gavaged with 500 mg/kg of FITC-dextran. The serum level of FITC-dextran was then measured in blood harvested 4 hours after administration.

### Total liver lipids

For hepatic lipid measurements, 100 mg of liver tissue was homogenized at 4°C in lysis buffer containing 50 mM Tris (pH 8.0), 150 mM NaCl, and 1% Triton X-100. Lipids were extracted from the total liver homogenate using the chloroform-and-methanol method [38].

### Histological assessment

Liver specimens were fixed in 10% neutral buffered formalin and embedded in paraffin. Histological sections (4 μm) were then stained with hematoxylin and eosin (H&E) or periodic acid-Schiff (PAS), coded, examined and graded by two independent investigators that were blind to the samples according to published criteria for magnitude analysis of steatosis [39] . Briefly, the degree of steatosis was graded 0–4 based on the average percent of fat accumulated hepatocytes per field at × 200 magnification (Grading 0 =<5%, 1 = 5~25%, 2 = 26~50%, 3 = 51~75%, 4 = >75%).

### Statistical analysis

Analyses were performed using the GraphPad Prism 5.3 software. The data are shown as the mean ± SEM. Comparisons between two groups were performed using Student’s *t*-test for unpaired data. Three or more group comparisons were carried out using one-way ANOVA followed by Student-Newman-Keuls multiple comparisons test. A *P* value less than 0.05 were considered significant.

## Additional files

Additional file 1:
**Analysis of hydric consumption among experimental groups used in the present study.**
Additional file 2:
**Composition of the high-fiber (HF) (10% pectin, modified AIN93M diet) and low fiber (LF) diets.**
