# Supplementary material for: Comparing the effects of acute alcohol consumption in germ-free and conventional mice: the role of the gut microbiota
Source: BMC Microbiol. 2014 Sep 16;14:240. doi: 10.1186/s12866-014-0240-4 (PMC4177591; doi:10.1186/s12866-014-0240-4)
Supplement: Additional file 2: — Composition of the high-fiber (HF) (10% pectin, modified AIN93M diet) and low fiber (LF) diets. [file 12866_2014_240_MOESM2_ESM.pdf]

Supplementary 2

Table 1. Composition of the high-fiber (HF) (10% pectin, modified AIN93M diet) and low fiber (LF) diets.

Ingredients	Per Kg of HF diet	Per Kg of LF diet
BHT	0.008 g	0.008 g
Choline Chloride	2.5 g	2.5 g
DL Methionine	1.8 g	1.8 g
AIN93 Vitamins	10.0 g	10.0 g
AIN93 Trace Minerals	35.0 g	35.0 g
Cellulose	50.0 g	50.0 g
Canola Oil	40.0 g	40.0 g
Sucrose	100.0 g	100.0 g
Dextrinized Starch	55.0 g	155.0g
Casein	140.0 g	140.0 g
Wheat Starch	365.692 g	365.692 g
**Pectin**	**100.0 g**	**0.0g**
H_2O	200 ml	200ml
